# Data on TOF-SIMS analysis of Cu^2+^, Co^2+^ and Cr^3+^ doped calcium phosphate cements

**DOI:** 10.1016/j.dib.2017.06.012

**Published:** 2017-06-13

**Authors:** Anja Henß, Martha Schamel, Uwe Gbureck, Michael Gelinsky, Anja Lode

**Affiliations:** a Institute of Physical Chemistry, Justus Liebig University of Giessen, Heinrich-Buff-Ring 17, 35392 Giessen, Germany; b Department for Functional Materials in Medicine and Dentistry, University of Würzburg, Pleicherwall 2, 97070 Würzburg, Germany; c Centre for Translational Bone, Joint and Soft Tissue Research, University Hospital Carl Gustav Carus and Faculty of Medicine of Technische Universität Dresden, Fetscherstraße 74, 01307 Dresden, Germany

## Abstract

This article contains data of time of flight secondary ion mass spectrometry (TOF-SIMS) analysis of brushite-forming calcium phosphate cements doped with biologically active metal ions. This data are related to the research article “Cu^2+^, Co^2+^ and Cr^3+^ doping of a calcium phosphate cement influences materials properties and response of human mesenchymal stromal cells” (Schamel et al., 2017) [1]. Cu^2+^, Co^2+^ and Cr^3+^ doped β-tricalcium phosphate precursor powders were used to prepare cement samples. The incorporation and distribution of the metal ions in the cement matrix was visualized by imaging mass spectrometry.

**Specifications Table**

**Table:** 

Subject area	*Physics, Chemistry*
More specific subject area	*Mass spectrometric monitoring*
Type of data	*Images and graphs*
How data was acquired	*Time of flight secondary ion mass spectrometry (TOF-SIMS)*
Data format	*Analyzed*
Experimental factors	*Monolithic cement samples prepared from Cu*^*2+*^*, Co*^*2+*^*and Cr*^*3+*^*doped β-tricalcium phosphate precursor powders*
Experimental features	*The samples were bombarded by Bi*_*3*_^*+*^*primary ions, secondary ions from the sample surface were emitted, collected by the analyzer and separated by their mass to charge ratio; distribution of the metal ions was imaged by rasterizing the primary ion beam over the sample surface*
Data source location	*Giessen, Germany*
Data accessibility	*Data with this article*

**Value of the data**

• TOF-SIMS is a high sophisticated method for surface analysis and enables mapping and 3D imaging of biomaterials [2], [3] with high sensitivity and high spatial resolution.
• TOF-SIMS data visualize the incorporation of metal ions in the cement matrix [1] by direct measurement of the samples.
• TOF-SIMS data provide additional information on the distribution of the metal ions in the cement matrix.

## 1 Data

**Fig. 1:**
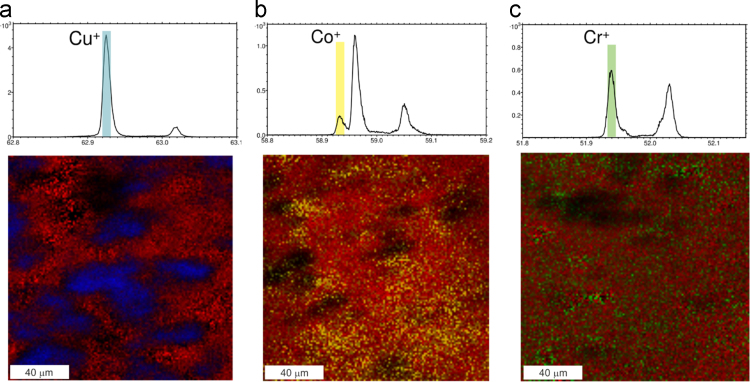
ToF-SIMS images and extracts from the corresponding mass spectra of the three different cement samples: (a) the Cu^2+^ modified cement, (b) the Co^2+^ modified cement and (c) the Cr^3+^ modified cement. The mass spectra prove the unambiguous assignment of the metal ion signals while the images provide information about the homogeneous distribution of (b) Co^+^ and (c) Cr^+^ and the clustering of (a) Cu^+^ in the cement. The images are overlays of the Ca_2_PO_4_^+^ signal in red and (a) Cu^+^ in blue, (b) Co^+^ in yellow and (c) Cr^+^ in green.

Data of TOF-SIMS analysis in this article provide information on the integration of three metal ions, Cu^2+^, Co^2+^ and Cr^3+^, in a calcium phosphate cement matrix. The distribution of Cu^2+^, Cr^3+^ and Co^2+^ in the crystalline matrix is visualized (Fig. 1).

## 2 Experimental design, materials and methods

Cement samples were prepared using β-tricalcium phosphate powder doped with 50 mmol Cu^2+^, Co^2+^ and Cr^3+^ per mol as described by Schamel et al. [1]. The TOF-SIMS analysis was done with a TOF-SIMS^5^ machine from the IONTOF GmbH Münster equipped with a Bismuth-cluster-source. Data evaluation was performed using the Surfacelab software version 6.5. For the measurement the samples were bombarded by Bi_3_^+^ primary ions under high vacuum conditions. Due to the impact of the primary ions secondary particles from the sample surface were emitted. The charged particles, or the so called secondary ions, were collected by the analyzer and separated by their mass to charge ratio, as all particles have the same kinetic energy. By rasterizing the primary ion beam over the sample surface, the distribution of each component can be imaged with high lateral resolution. A more detailed description of the method can be found elsewhere [2]. For the analyses regions of 150 × 150 μm^2^ were measured within 50 scans using the spectrometry mode which provided a mass resolution *m*/Δ*m* at (C_2_H_5_^+^) *m*/z=29,04 of ~4000 and a lateral resolution of less than 10 μm. The spectra were calibrated using the following signals: H^+^, H_2_^+^, C^+^, CH_3_^+^, C_2_H_5_^+^, C_3_H_3_^+^. The images given in Fig. 1 are plotted as overlays where Ca_2_PO_4_^+^ is shown in red, Cu^+^ in blue, Co^+^ in yellow and Cr^+^ in green color. As ToF-SIMS is not a quantitative method by itself, these analyses do not reveal information about the content of metal ions in the different samples.
